# A comparison of treatment modalities for nasal extranodal natural killer/T-cell lymphoma in early stages: The efficacy of CHOP regimen based concurrent chemoradiotherapy

**DOI:** 10.18632/oncotarget.13614

**Published:** 2016-11-25

**Authors:** Jianzhong Cao, Shengmin Lan, Liuhai Shen, Hongwei Si, Ning Zhang, Hongwei Li, Ruyuan Guo

**Affiliations:** ^1^ Department of Radiation Oncology, The Cancer Hospital of Shanxi Province, Taiyuan, Shanxi Province, 030013, China; ^2^ Department of Nuclear Medicine, The First Affiliated Hospital of Anhui Medical University, Hefei, Anhui Province, 230022, China

**Keywords:** nasal extranodal natural killer/T-cell lymphoma, concurrent chemoradiotherapy

## Abstract

This study was designed to evaluate the efficacy of several treatment modalities, including CHOP based concurrent chemoradiotherapy (CCRT), for the patients with stage IE or IIE nasal extranodal NK/T-cell lymphoma (nasal ENKL). The cases were retrieved between 2000 and 2010 (n=94), and were followed to the end of February 2016. The patients were grouped into A (chemotherapy alone; CT alone), B (sequential treatment) and C (CCRT). For those with efficacy evaluation for overall treatment (n=90), CR was attained in 60.0% (18/30), 69.8% (30/43) and 76.5% (13/17) patients in the group A, B and C, respectively. The 5-year OS rate was 35.2%, 41.9% and 70.6% in the group A, B and C, respectively. For patients with early stage diseases (IE and IIE), the ECOG performance status and the Ann Arbor stage were significant prognostic factors for both OS and PFS. Among the stage IE patients, besides the ECOG performance status, three prognostic factors which related to treatments (treatment modalities, efficacy of initial and overall treatment) were significant against OS or PFS. In conclusion, compared to chemotherapy alone and sequential treatment, nasal ENKL patients in early stages, especially stage IE, benefit the most from CHOP based concurrent chemoradiotherapy.

## INTRODUCTION

Nasal extranodal natural killer/T-cell lymphoma (nasal ENKL), which strongly associated with Epstein–Barr virus infection, is relatively common in Asian and Latin American countries [1]. Although the patients initially have good performance status and low international prognostic index (IPI) scores [2], nasal ENKL is the most aggressive subtype of non-Hodgkin's lymphoma [3].

Because of the frequently expressed multidrug resistance (MDR) associated p-glycoprotein (P-gp), nasal ENKL patients usually do not efficiently respond to chemotherapy (CT), especially CHOP (cyclophosphamide, doxorubicin, vincristine and prednisone) or CHOP like regimens [4]. For the localized diseases in early stages, radiotherapy (RT) is considered as the front-line treatment [5], and usually produces a more rapid response and a higher complete remission rate (CR) [1]. However, RT alone is insufficient to improve overall survival (OS), and needed to be combined with CT in an appropriate sequence [6]. Among the combined treatment modalities, concurrent chemoradiotherapy (CCRT) using MDR- unrestricted agents [7] and radiation sensitizers [8, 9] can improve the CR rate to 73.3% and 77%, respectively [10]. However, the optimal treatment of early stages nasal ENKL remains to be determined.

To evaluate the relatively rare disease, it is not easy to perform a prospective trial. Therefore, we performed this retrospective study to compare the efficacy of CCRT, sequential treatments and CT alone for nasal ENKL in early stages.

## RESULTS

### Patient characteristics and treatment

Between 2000 and 2011, a total of 99 patients were enrolled, and five cases were excluded for lost to follow up. Patient characteristics are listed in Table 1. Median age of the patients was 42.0 years (range 14 to 74 y), and the majority were younger than 60 years (n=85, 90.4%). There were 34, 43 and 17 patients in the group A (CT alone), B (sequential treatment) and C (CCRT), respectively. The percentages of patients with an ECOG performance status of 0-1 were 67.6% (23/34), 86.0% (37/43) and 88.2% (15/17) in the group A, B and C, respectively. In the group B, 11 patients were treated with RT followed by CT (RT+CT), and 32 patients were treated with CT followed by RT (CT+RT).

**Table 1:** Patient characteristics

Characteristic		Group A	Group B	Group C	*p*	All n (%)
Gender	Male	14(82.4)	33(76.7)	27(79.4)		74 (78.7)
	female	3(17.6)	10(23.3)	7(20.6)	0.947	20 (21.3)
B symptoms	Absent	10(58.8)	25(58.1)	23(67.7)		58 (61.7)
	Present	7(41.2)	18(41.9)	11(32.4)	0.642	36 (38.3)
Waldeyer's ring	Absent	14(82.4)	35(81.4)	25(73.5)		74 (78.7)
	Present	3(17.6)	8(18.6)	9(26.5)	0.712	20 (21.3)
Local invasion	Absent	9(52.9)	14(32.6)	19(55.9)		42 (44.7)
	Present	8(47.1)	29(67.4)	15(44.1)	0.094	52 (55.3)
LDH level	Normal	15(88.2)	37(86.0)	30(88.2)		82 (87.2)
	Abnormal	2(11.8)	6(14.0)	4(11.8)	1.000	12 (12.8)
Ann Arbor stage	IE	16(94.1)	36(83.7)	20(58.8)		72 (76.6)
	IIE	1(4.5)	7(16.3)	14(41.2)	0.007	22 (23.4)
New staging	Limited IE	9(52.9)	10(23.3)	9(26.5)		28 (29.8)
	Extensive IE	7(41.2)	26(60.5)	11(32.4)		44 (46.8)
	IIE	1(5.9)	7(16.3)	14(41.2)	0.007	22 (234)
IPI score	0	9(52.9)	34(79.1)	28(82.4)		71 (75.5)
	1	8(47.1)	8(18.6)	6(17.6)		22 (23.4)
	2	0(0)	1(2.3)	0(0)	0.078	1 (1.1)
ECOG performance status	0-1	15(88.2)	37(86.0)	23(67.6)		75 (79.8)
	≥2	2(11.8)	6(31.6)	11(32.4)	0.118	19 (20.2)

Among the group A, B and C, except for the new staging system (*x*^2^=13.694, *p*=0.007) and the Ann Arbor stage (*x*^2^=9.557, *p*=0.007), other patient characteristics did not exist significant difference, including IPI (*x*^2^=7.106, *p*=0.078), age (F=2.066, *p*=0.133), gender (*x*^2^=0.237, *p*=0.947), and ECOG performance status (*x*^2^=4.442, *p*=0.118). The differences among the groups were mainly from the group A, which enrolled more stage IIE patients than other groups. Between the group B and C, the new staging system (*x*^2^=5.202, *p*=0.074) and the Ann Arbor stage (*x*^2^=1.140, *p*=0.286) did not have significant difference.

Radiotherapy (n=60) was given by the Varian 6-MV linear accelerator (median radiation dose 50Gy, range 30-70Gy). The median dose of group B and C was both 50 Gy, and was higher than 50 Gy in 13/43 and 6/17 patients (*x*^2^=0.014, p=0.907), respectively. In the group B, 7/34 patients accepted intensity modulated radiotherapy (IMRT), and no patients in the group C accepted the treatment.

For the treatments of CT (n=94), 77 patients were administrated 1-6 cycles (median: 4 cycles) of CHOP (or CHOP like) regimens (n=21, 39 and 17 in the group A, B and C). Other patients accepted 1-6 cycles (median: 4 cycles) of BACOP (bleomycin, adriamycin, cyclophosphamide, oncovin and prednisone, 11 and 2 patients in the group A and B), and 2-13 cycles (median: 5.5 cycles) CAV (cyclophosphamide, doxorubicin and vincristine, 2 and 2 patients in the group A and B). All patients in group C accepted 1-8 cycles of CHOP regimens (median 3.5).

### Patterns of treatment failure

Among the patients who failed to the treatments (n=37), the patterns of failures were loco-regional and distant lymph node failure (n=20) and systemic failure (n=9), and 8 patients encountered both lymph node and systemic failure. Extranodal failure were observed in bone marrow (n=6), skin (n=5), lung (n=2), testis (n=2), hypopharynx (n=2), brain (n=1), and colon (n=1).

When the disease recurred or progressed, salvage treatments were chemotherapy. The regimens included CHOP (or CHOP like, n=9), EPOCH (etoposide, doxorubicin, vincristine, cyclophosphamide and prednisone, n=6), IMVP-16 (ifosfamide, etoposide and methotrexate, n=13), DHAP (dexamethasone, cytarabine, and cisplatin, n=5) and GEMOX (gemcitabine and oxaliplatin, n=4) regimens.

### Treatment toxicity

All treatments were tolerable in most patients. The most common toxicity was neutropenia which developed from chemotherapy, and the mucositis from radiotherapy. In Table 2, only >2 radiation mucositis of group C is significantly higher than other groups. In the group A and B, four and three patients died of neutropenia associated infection, respectively. One patient in the group C died of treatment unassociated cerebral infarction.

**Table 2:** Toxicity of treatments

	Group A		Group B		Group C		*x*^2^
Toxicity	≤2	>2	≤2	>2	≤2	>2	
**Hematologic**							
Neutropenia	20(58.8)	14(41.2)	27(62.8)	16(37.2)	10(58.8)	7(41.2)	0.008
Anemia	32(94.1)	2(5.9)	38(88.4)	5(11.6)	13(76.5)	4(23.5)	3.214
Thrombocytopenia	32(94.1)	2(5.9)	41(95.3)	2(4.7)	14(82.4)	3(17.6)	1.554
**Non-hematologic**							
Nausea	32(94.1)	2(5.9)	39(90.7)	4(9.3)	15(88.2)	2(11.8)	0.561
Vomiting	34(100)	0(0)	43(100)	0(0)	16(94.1)	1(5.9)	2.764
Diarrhea	34(100)	0(0)	43(100)	0(0)	17(100)	0(0)	-
Anorexia	34(100)	0(0)	43(100)	0(0)	17(100)	0(0)	-
Constipation	34(100)	0(0)	43(100)	0(0)	17(100)	0(0)	-
Radio-mucositis	34(100)	0(0)	43(100)	0(0)	13(76.5)	4(23.5)	11.426^*^
Radiodermatitis	34(100)	0(0)	43(100)	0(0)	16(94.1)	1(5.9)	2.764

^*^: p<0.05; **: p<0.01.

### Treatment response

**Figure 1:**
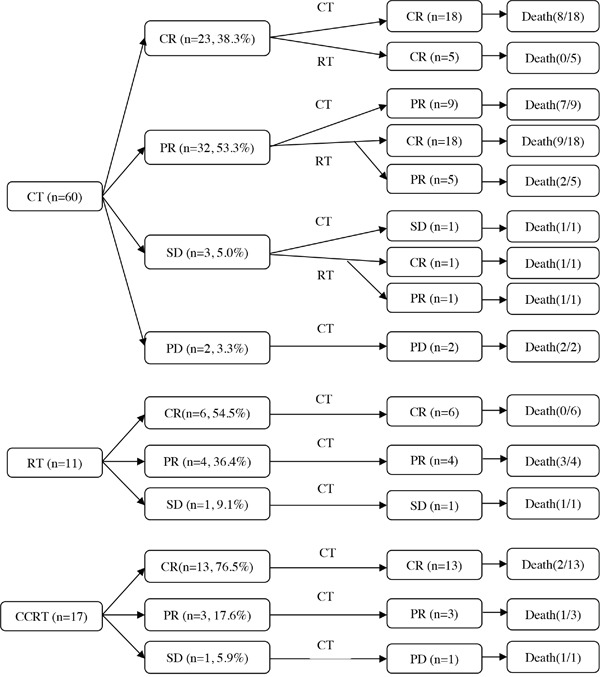
Treatment characteristics of patients (n=88) after initial and overall treatment

Six patients did not have efficacy initial and/or overall treatment evaluations: 2 patients without initial evaluation achieved PR after overall treatments (group B, 1/2 died), and 4 patients were not evaluated after both initial and overall treatments (group A, 2/4 died). For patients with initial treatment evaluations (n=88, Figure 1), CR were attained in 60.0% (18/30), 26.8% (11/41) and 76.5% (13/17) patients in the group A, B and C, respectively. For patients with overall treatment evaluations (n=90), CR was attained in 60.0% (18/30), 69.8% (30/43) and 76.5% (13/17) patients in the group A, B and C, respectively. Overall response rates (ORR) to overall treatments were 90.0% (27/30), 97.7% (42/43) and 94.1% (16/17) in the group A, B and C, respectively. In the group B, CR rates to CT+RT and RT+CT were 75.0% (24/32) and 54.5% (6/11), respectively. Among patients accepted CT+RT, CR rate to initial treatment for chemotherapy was only 15.6% (5/32).

### Survivals and prognosis

By the end of February 2016, survival and local recurrence information were collected (n=94). The median observation times were 36.6 (range 6.7-179.5 months), 27.3 months (range 5.9-178.6) and 63.4 months (range 11.9-151.8) for the group A, B and C, respectively. Tumor progression occurred within a median time of 49.6 months (range 9.1–97.3 months). In the group A, B and C, recurrence was observed in 23/34 (67.6%), 8/43 (18.6%) and 3/17 (17.6%) patients, respectively. Forty-two patients died within 6.0 to 178.0 months. There were 20/34 (58.8%), 18/43 (41.9%) and 4/17 (23.5%) deaths in the group A, B and C, respectively.

**Figure 2:**
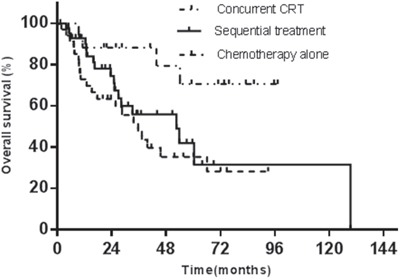
The initial treatment modalities against OS

**Figure 3:**
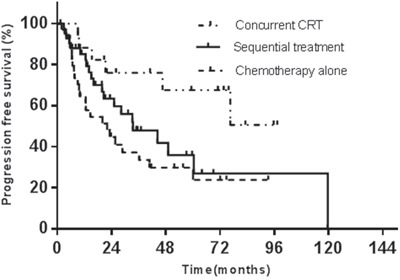
The initial treatment modalities against PFS

5-year OS and progression-free survival (PFS) rates are listed in Table 3. Among the treatment groups, both OS (*x*^2^= 7.086, *p*=0.029) and PFS (*x*^2^= 7.378, *p*=0.025) had significant difference. In the multivariate Cox regression (conditional forward) against OS, the ECOG performance status (odds ratio 2.2, 95% CI 1.4~3.5, p=0.001) and the Ann Arbor stage (odds ratio 2.9, 95% CI 1.5~5.6, p=0.001) showed statistical significance (Figure 2). In the Cox regression against PFS, the ECOG performance status (odds ratio 2.3, 95% CI 1.5~3.6, p=0.000) and the Ann Arbor stage (odds ratio 1.8, 95% CI 1.2~2.7, p=0.002) had statistical significance (Figure 3).

**Table 3:** Survival analysis

		N	5-year OS(%)	Median (months)	*p*	5-year PFS(%)	Median (months)	*p*
**Gender**	Male	74	45.8	53.9	0.479	40.7	36.2	0.559
	Female	20	42.8	45.6		39.8	33.2	
**Age (years)**	≤60	85	43.2	52.6	0.580	38.0	33.2	0.526
	>60	9	66.7	NA		66.7	76.6	
**Radiation**	<50	24	45.2	53.9	0.858	43.4	44.3	0.200
**Dose (Gy)**	≥50	36	58.6	NA		67.3	NA	
**B symptoms**	Absent	58	42.5	52.6	0.354	37.9	28.3	0.287
	Present	36	47.1	45.6		44.9	40.8	
**Waldeyer’s**	Absent	74	47.5	54.2	0.712	43.1	33.5	0.675
**ring**	Present	20	39.7	52.6		31.5	36.2	
**Local**	Absent	42	50.8	66.1	0.442	45.0	33.5	0.500
**invasion**	Present	52	38.7	45.6		36.0	36.2	
**LDH level**	Normal	82	48.7	54.2	0.490	43.1	36.2	0.405
	Abnormal	12	0	33.7		0%	25.6	
**Ann Arbor**	IE	72	57.5	129.4	0.000	49.9	49.0	0.000
**stage**	IIE	22	10.9	16.1		11.3	16.1	
**New staging**	Limited IE	28	73.8	NA	0.000	63.6	76.6	0.000
	Extensive IE	44	44.0	53.9		38.9	40.8	
	IIE	22	10.9	16.1		11.3	16.1	
**IPI score**	0	71	47.4	56.8	0.885	41.9	61.6	0.842
	1	22	37.5	45.0		33.7	40.9	
	2	1	0.0	12.0		0.0	12.0	
**ECOG performance**	0-1	75	56.4	66.1	0.000	49.2	49.0	0.000
**status**	≥2	19	7.2	16.5		6.3	12.5	
**Initial**	CT	66	37.7	37.4	0.048	30.1	28.8	0.038
**treatment**	RT	11	58.4	NA		24.2	14.0	
	CCRT	17	70.6	NA		67.6	NA	
**Treatment**	CT only	34	35.2	35.7	0.029	29.8	22.0	0.025
**modalities**	Combined therapy	43	41.9	52.6		36.0	33.5	
	CCRT	17	70.6	NA		67.6	NA	

NA: Not arrive.

In the group B, for CT+RT and RT+CT, 5-year OS rates were 41.3% and 58.4%, and 5-year PFS rates were 39.0% and 24.2%, respectively. The 5-year OS and PFS did not have significant differences between CT+RT and RT+CT treatment (p=0.593 and p=0.140). The median overall survival times for CT+RT and RT+CT were both 52.6 months, and the median progression-free survival times for the two treatment modalities were 44.3 and 32.6 months, respectively.

### Statistical analysis for stage IE disease

Only limited cases in stage IIE were enrolled (14, 7 and 1 patients in the group A, B and C), and none of the influential factors was significant against OS or PFS.

There were 20, 36 and 16 stage IE patients in the group A, B and C, respectively. The 5-year OS rates were 50.7%, 53.9% and 75.0% (*x*^2^=3.599, p=0.165), and the 5-year PFS rates were 40.0%, 44.7%, and 71.8% (*x*^2^=3.972, p=0.137) in the group A, B and C, respectively. The multivariate Cox regression test against PFS indicated that the treatment modalities (odds ratio 2.1, 95% CI 1.3~3.4, p=0.003) and the efficacy of overall treatment (odds ratio 3.0, 95% CI 2.0~4.6, p=0.000) were significant factors. In the Cox regression test against OS, the ECOG performance status (odds ratio 2.2, 95% CI 1.1~4.2, p=0.022) and the efficacy of initial treatment (odds ratio 1.6, 95% CI 1.0~2.5, p=0.030) were significant factors.

## DISCUSSION

In this study, the most characteristics of early stages ENKL patients were balanced between the treatment groups, except that the new staging system and the Ann Arbor stage of group A (enrolled more stage IIE patients) were different from that of other groups. Our results indicate that, among all patients, the ECOG performance status and the Ann Arbor stage are both significant against OS or PFS. Among the stage IE patients, besides the ECOG performance status, three factors (treatment modalities, efficacy of initial and overall treatment) related to therapy are significant against OS or PFS. Because CHOP based concurrent chemoradiotherapy can produce a higher CR rate and a longer 5-year OS (or 5-year PFS) rate than chemotherapy alone or sequential treatment, the CCRT treatment is a better modality for early stages nasal ENKL patients, especially for those in stage IE.

In the past, RT alone was the first-line treatment of nasal ENKL (5-year OS rate about 40%) [11]. After it was classified as a lymphoma, chemotherapy became the first-line treatment modality [12]. Although some patients could be cured by CHOP (or CHOP-like) based chemotherapy [13], nasal ENKL frequently fails to respond to the regimens [14]. In the study from Wang et al [15], CHOP based chemotherapy, as an induction treatment, could produce a CR rate of 31.8%. In our patients treated with CT alone, additional chemotherapies could not efficiently convert them from PR to CR. This is consistent with the consensus that some nasal ENKL patients can benefit from the initial treatment of CHOP regimens, but not additional ones [4]. New regimens, for example L-asparaginase and gemcitabine regimen, were reported to be effective for nasal ENKL patients (2-year OS rate: 87.1%) [16–18]; however, the regimens needed to be evaluated further.

Although radiotherapy usually produced a rapid treatment response, only 11 patients in this nasal ENKL cohort were initially treated by radiotherapy, and achieved a CR rate of 54.5% (6/11). After initial treatment of chemotherapy, RT could convert 18/23 (78.3%) patients from PR to CR. Additionally, although poor responders to CT tended to accept RT in our hospital, the 5-year OS rates to CT+RT and RT+CT (38% and 58%) were comparable to other studies [12], and were higher than CT alone (35.2%). Above all, our paradigm interfered with treatment selection more or less, and the efficacy of RT should be further evaluated.

Currently, CCRT had been regarded as a treatment option for localized diseases [9]. Using MDR-unrestricted agents [8], radiation sensitizers [7] or shrinking-field radiation strategy [19] to induce antitumor effect, a higher 5-year OS rate could be achieved (73%, 78.6% and 80%, respectively). In our study, CHOP based CCRT could even produce a comparable 5-year OS and PFS rate (70.6% and 67.6%). Furthermore, except radiation mucositis, the toxicity profiles of CCRT for this cohort were also comparable to other groups, and are consistent with these CCRT studies. Therefore, it seems that, for patients with nasal ENKL in early stages, the initial treatment of CHOP based CCRT is efficacious, and is less influenced by the frequently expressed P-glycoprotein (P-gp).

Our results can be explained by the study on advanced breast cancer [20]. P-gp, an ATP dependent membrane transporter which pumps cytotoxic drugs out of cells, is coded by the MDR1 (ABCB1) gene. MDR1 transcription can be started at 2 promoters: a major downstream promoter (DSP) and a minor upstream promoter (USP). Although transcripts from USP are associated with translating polyribosomes, their low concentration makes the amount of P-gp insufficient to affect the response of cells to an initial chemotherapy [20].

Additionally, among patient characteristics, only the Ann Arbor stage and the new stage were not balanced among the treatment groups. However, according to our results, the two stage systems would obviously affect the prognosis of the patients. When stratified by the Ann Arbor stage, 5-year OS and PFS rates were dramatically decreased in stage IIE patients (Table 3). Furthermore, stratified IE patients with the new stage system, the two rates for extension disease were lower than that of limited disease (Table 3). These results supported the prognostic value of the two stage systems, and coincided with other studies [21, 22]. Therefore, it is possible that our results could be influenced by the poor prognosis of stage IIE patients, especially the results from group A (recruited more stage IIE patients). However, our results from stage IE patients confirmed the results from all patients, and indicated CCRT is superior to other treatment modalities. Because only one stage IIE patient was in the CCRT group, the response of stage IIE patients to CHOP based CCRT should be verified in future.

In conclusion, compared to chemotherapy alone and sequential treatment, patients with nasal ENKL in early stages, especially stage IE, benefit the most from CHOP based concurrent chemoradiotherapy. This treatment strategy can attain a comparable CR rate to other CCRT studies.

## PATIENTS AND METHODS

### Patients

Nasal ENKL cases in stage IE and IIE (Ann-Arbor stage [23]) were retrieved from the files of the Cancer Hospital of Shanxi Province between 2000 and 2011. All patients were diagnosed according to the morphological and immunohistological criteria recommended by the World Health Organization classification [24]. Additionally, the patients were scored and restaged according to the international prognostic index (IPI) and the new staging system [21], respectively. Our protocol was approved by the ethics committee at the Cancer Hospital of Shanxi Province, and signed informed consent forms were obtained from all participants, who were consented to be followed.

Because there was no standard treatment for nasal ENKL, the paradigm of our hospital was not accordant, and CHOP regimens were most frequently administrated ones. Generally, for classifying as a lymphoma, chemotherapy was the first-line treatment, and only the poor responders would accept radiotherapy. After the study in 2006 from Li et al [25], more nasal ENKL patients accepted radiotherapy than before (75% vs 51%, Fisher's exact test p=0.022), but still not the first choice.

From medical records or by telephone, the patients were followed to the end of February 2016, and were grouped into A (CT alone), B (sequential treatment) and C (CCRT) according to treatment modalities. The radiation fields of limited stage IE diseases included the bilateral nasal cavity, bilateral ethmoid sinuses, and ipsilateral maxillary sinus. For extensive stage IE patients, the fields were extended to adjacent involved structures. For stage IIE diseases, bilateral cervical lymph node areas were also covered. Additionally, fields for Walder ring included the ring, adjacent involved organs or structures, and cervical lymph nodes.

Before treatments, the Eastern Cooperative Oncology Group (ECOG) performance status was evaluated. According to the revised response criteria for malignant lymphoma [26], response to initial treatment (CT, RT, or CCRT) was evaluated by physical examinations and computerized tomography obtained 3 to 4 weeks after the completion of treatments. Response to overall treatments was assessed 3 to 4 weeks later. In the CCRT arm, RT and CT began on the same day, and response was also assessed 3 to 4 weeks later.

Tumor responses were classified into complete remission (CR), partial remission (PR), stable disease (SD) and progressive disease (PD) based on a radiographic review of MRI or computed tomography.

### Statistical analysis

PFS was measured from the day treatment began to the first event of either recurrence or death. OS was measured from the day of diagnosis to death from any cause. All data was inputted into the SPSS statistical software (version 10.01, SPSS Inc, Chicago, IL). Kaplan–Meier and Log-rank test were applied to calculate the survival rates and to compare survival curves, respectively. The Cox regression analysis was used to find out the significant prognostic factors for PFS or OS. p<0.05 was considered as the significant level.
